# Ten Simple Rules for Developing a Successful Research Proposal in Brazil

**DOI:** 10.1371/journal.pcbi.1005289

**Published:** 2017-02-02

**Authors:** Dyoni M. de Oliveira, Marcos S. Buckeridge, Wanderley D. dos Santos

**Affiliations:** 1 Laboratory of Plant Biochemistry, Department of Biochemistry, State University of Maringá, Maringá, Paraná, Brazil; 2 Department of Botany, Institute of Bioscience, University of São Paulo, São Paulo, São Paulo, Brazil

## Introduction

Writing well is fundamental to publishing and having a successful scientific career [1], and being able to write a good research proposal is critical for obtaining financial support [2]. In emerging economies, such as Brazil, it is necessary to confront drawbacks not encountered in high-income countries [3]. The developing world has growing investments in science, technology, and innovation in many areas [4–6], including computational biology [7]. These investments have produced positive results in scientific quality in developing countries [8]. Although this is remarkably positive, the emergence of high-level research groups creates a highly competitive environment. We suggest a roadmap of ten simple rules for writing a consistent and convincing research project, which may be useful for researchers in Brazil and other emerging economies. There are several funding agencies in Brazil, and two of them—the National Council for Research Development (CNPq) and the São Paulo Research Foundation (FAPESP)—are used as examples of how proposals can be better adjusted in order to be successful. The latter represents the state funding agencies. Our ten rules will consider these agencies as the generic targets of proposals. When describing the ten rules below, we consider applications for research grant proposals and for MSc and PhD fellowships.

## Rule 1: Define the Problem Clearly

In general, the most important part of a research project is to precisely define the problem to be investigated. If you wish to ask for financial support for your research, it is imperative to attest that your interest is in line with research supported by the funding calls available. Some calls are generic and flexible, such as the Universal Call from the CNPq in Brazil. It is not essential to fit into specific calls, but it is certainly easier to swim downstream whenever possible. The relevance and originality of your research targets and, of course, internal coherence (between targets and methods) have a significant impact on the value of the project. An extensive and updated review of the relevant literature can guarantee the originality of your targets, averting as much as possible the risk of your findings being published by another group while your project is still ongoing. In this case, the risk is producing good results without relevance to your field. Although the originality and relevance of a proposal can be ensured, the relevance of your findings is unpredictable.

Originality is usually inversely proportional to risk. When an idea is proposed, the level of novelty may lead a reviewer to find the project too risky. When preparing your proposal, it is therefore important to describe the risks very clearly. Brazilian reviewers tend to be quite conservative, and even a low risk level can be considered too much risk. When reviewers of FAPESP, for example, are completing the review form, there are boxes at the end that have to be ticked. If a box like “Very Good with Minor Deficiencies” is ticked, the coordinator (the level above a reviewer) who will make the final decision may hesitate to approve the proposal. Authors thus have to be very careful and clearly explain the risks related to the project in order to minimize the possibility of the reviewer ticking the boxes that point out deficiencies. Because this is a cultural problem related to the reviewers rather than to the applicants, it is very important that the applicant display preliminary results that clearly and elegantly show the reviewer that the risks are manageable. This is difficult and demands hard and careful thinking, but it is the only way to change the conservative culture of Brazilians into one that incorporates a more open and braver view of the work in science.

## Rule 2: Formulate Falsifiable Hypotheses and Include Preliminary Data

Sometimes, you can summarize your research as a precise and complete survey of data; however, when studying complex systems (such as living beings), measuring everything might not be feasible or convenient. It can therefore be useful to formulate a hypothesis that you can test with a number of experiments. You must formulate the hypothesis as an affirmative, clear, and concise sentence (e.g., “The volume of the liquid water is directly proportional to the temperature”). This statement must express an up-to-date possibility based on a systematic review of scientific knowledge on the defined theme; however, you cannot know beforehand whether your hypothesis is really correct (as in the example above, which we currently know to be wrong). The important thing here is to ensure that your hypothesis is testable under the actual conditions you have or have access to (physical, financial, and human resources) so that you can develop plausible experiments to test it. In low-income countries, being creative in the proposition of accessible methodologies for testing a hypothesis is especially critical, as we discuss in Rule 5.

Often, the hypothesis of a research project arises as a result of previous observations, and presenting the preliminary data might provide crucial support for your hypothesis. The preliminary data will also help you to effectively convince a reviewer that you have the technical and scientific expertise to carry out the work as proposed[2].

Again, try to prepare the text so that the reviewer concludes, after reading the project, that what you want to do is indeed science and that it is more than that: it is good science that advances knowledge.

## Rule 3: Establish Clear Objectives

After formulating your hypothesis (or hypotheses), you should establish a clear and explicit goal, the necessity of which is exemplified in the excerpt below:

*Finding herself lost in Wonderland*, *Alice asked to the Cheshire Cat*: *“Would you tell me*, *please*, *which way I ought to go from here*?*” “That depends a good deal on where you want to get to*,*” the cat replied*. *"I don't much care where—" said Alice*. *"Then it doesn't much matter which way you go*,*” said the cat* [9].

You can find much by chance or serendipity; however, what you discover by chance will not necessarily be the same thing you were looking for. In a project, you want to convince others to trust your goals and that you are competent. A clear goal will guide the choice of the methodologies that you will use to get there. Objectives underlie an experimental design and can serve as a basis for performance evaluation and a change of strategy, when necessary. During the execution of the research, you will probably have to divide your attention across many tasks: classes, paperwork, other projects, supervisions, and so on. A clear statement of the objectives will remind you and your collaborators of where are you going and how you intend to get there.

Most reviewers are busy scientists, and they have to perform a great deal of administration along with their scientific work. In some cases, they will read the objectives (and the title and abstract) more carefully than other parts of the project. Therefore, be absolutely sure that you are describing your objectives in a simple way.

## Rule 4: Estimate the Duration and Requirements of Experimental Procedures Carefully

If your goals define the specific procedures you will follow during the research, the reverse is also true. The design of your experimental approach will also help you define the goals of your proposal. To reach specific goals, it is important to gain access to the necessary in-house facilities or invite external collaborators, as previously discussed in Ten Simple Rules [10]. If you have no expertise, this can be crucial. Talk to experienced researchers about the techniques you have in mind. Ask how long procedures specifically require, and be careful about laconic answers—especially from your mentor! They can reflect optimistic expectations or the desire to obtain results without thinking deeply about realistic deadlines. Be sure that the time required to carry out experimental procedures is compatible with the maximum period established by the funding call. It is important to know as much as possible about experimental methodologies in order to avoid a design that is impracticable within the timeframe and with the resources available. Calculate the required time, allotting sufficient time for replanning. Bear in mind that scientific research is full of unpredictable mishaps (but also serendipity), and thus, it is important to evaluate the possible risks of things that do not go well. By identifying these risks, you can attempt to avoid them when managing your research. The funding agencies and especially private funders expect you to fulfil what you promised in the proposal, even after the deadline (and in this case, without additional resources!), and thus, it is always wise to promise the minimum necessary to achieve your goals. In Brazilian science, this is the most important failing point in proposals. Reviewers are usually not aware of those failures, however. Proposals in Brazil rarely include a schedule showing clearly when each milestone of the project should be reached, by whom it will be produced, and how the different tasks are associated with the objective of the project. However, this is one of the most important parts of the project because it gives the reviewer a clearer idea about the feasibility of the proposal. Your project may be original, the objectives may be clear, and the methodology choices may be appropriate, but if you do not construct a framework of tasks and resources (people and money) that are clearly coordinated, the reviewer will not be able to evaluate the feasibility of your project. Brazilian funding agencies usually fund a relatively small percentage of the proposals submitted. Final decisions are also made in a comparative review, in which a board of reviewers may decide together who will be approved. If your competitors have a more detailed schedule, they will thus have an advantage, and your proposal is more likely to be turned down.

## Rule 5: Explain the Methodologies for the Goal, to Demonstrate That You Can Carry out the Research

Provide methodological descriptions that best fit your needs, your knowledge, and your financial reality. Take special care not to write methodologies that are incomplete or inapplicable to your particular case. It is common to find inconsistencies in proposals due to the “copying and pasting” of methodologies from other proposals. A zealous reviewer may require correction, and you may find yourself in a difficult situation or be asked to correct your work, particularly if you have to defend your proposal in public. Remember that there are two kinds of knowledge: tacit and explicit. Written methodologies usually hide important details that belong to the domain of tacit knowledge. You learn the tricks of the trade only by practicing and training with an experienced researcher. If you only have access to explicit knowledge to perform an experiment, you will probably make mistakes. The person evaluating your proposal, who is usually specialized in the field, might consider this restriction by consulting your curriculum vitae. You can lessen the potentially negative impact of this problem through careful planning, which allows a surplus of time for establishing a protocol [11].

Some scientists and reviewers think that the methodology is the most important part of a proposal, so be certain that you are using (1) the right methods for the purpose of each experiment and (2) a currently accepted methodology. This does not always mean that you should only use the most advanced technology. Reviewers usually base their evaluation on the basis of a trade-off between the novelty of the method and the adequacy of using it, especially in light of how much money you are going to spend to perform the experiments.

## Rule 6: Clearly Define the Tasks, People in Charge, and Costs in Your Research Proposal

In order to answer a scientific question, it may be necessary to complete a series of goals and perform a series of experiments. It is thus useful to clearly define the following points for each goal: (1) What are the dates for initiating and finishing the experiment? (2) Who will carry out the experiment (in the case of a group)? (3) How much will the experiment cost? (4) How will you assess the research progress? (5) What are the critical risks? (6) How might you deal with severe problems? Even with good maintenance, equipment might fail, or a technician become unavailable. Try not to underestimate the deadline required for a crucial task, and, if possible, identify spare facilities/specialists to whom you can resort, if necessary.

## Rule 7: Preventing the Unpredictable: Establish a Flexible Schedule

The result of one goal may be essential for the start of another. It is thus advisable for you to outline the best order in which each task must be executed. Nonetheless, remember that the schedule works as a possible way to execute all necessary tasks in your proposal. Not everything you plan must happen exactly as you originally conceived it, and it is natural for the schedule to undergo changes throughout the project development. As such, a good schedule must be flexible enough to accommodate the unpredictable obstacles you will face.

If you have a well-constructed schedule, this could be where the reviewer will look very carefully to find inconsistencies and point them out as a deficiency. If the percentage of approval is low, the probability is that reviewers will start reading your proposal by looking for deficiencies. When they find something, they will consider this as an inconsistency in contrast with a top-level ideal proposal. You should thus expect to be penalized for every small mistake found in your proposal, and a complex schedule is somewhere a reviewer may find many problems, thus turning down your proposal.

## Rule 8: Justify the Benefits Your Research Will Provide

An exhaustive survey of the relevant literature will help you introduce the field and convince reviewers why the problem you chose deserves attention. If you are sure your research program is unique and relevant, prove it to the experts who will judge your proposal, presenting a complete state-of-the-art picture. Be parsimonious, however, with words, and do not lose focus. Before writing, establish a briefing with the necessary information. Organize the ideas as an inverted pyramid, from general information about the field to the specific area that your work will address. This will make it easy for the reader to follow your reasoning and to understand the focus of your research.

After the introductory context, it is important to stress to the reader why your work is important. Emphasize the practical advantages (technological) that may result from your research and the importance that these results may have in overcoming the knowledge gaps mentioned in the introduction. This is the time to provide a convincing support for the relevance and importance of your research project. A list of scenarios resulting from your research can facilitate appreciation by the reader of the possible impact of your proposal as a whole.

Currently, there is a clear trend for applications to note the societal implications of the research proposed, so it is quite important to explain the main connections between the results you will produce and the benefits they will bring to society. This issue is more important to the higher-level members of the funding agency—scientists who design and maintain the general policies of science for the country—than to the reviewer. If your proposal is considered excellent in all the items above, but your explanation of the benefits to the country or region is not clear, your proposal might be turned down if competition is tight.

## Rule 9: Write a Good Title and Abstract

The title must inform the reader about the scope of the project. It is common in scientific literature to have the title briefly describe the issue addressed in the work (article or project). Avoid the use of adverbs and scientific nomenclature, which are not strictly necessary. Remember that scientists are attracted by intelligence. A creative title can arouse empathy in the reviewer.

An abstract is optional; however, it facilitates reading and understanding the general idea of the proposal. Although you must avoid prolixity at all costs, depending on the complexity of the theme, the introduction may become long and complex. This may mislead the reader if you do not properly formalize the focus of the work in an abstract. Together, the title and the abstract are good opportunities to put forward your idea. Most reviewers will make their initial decisions about whether to approve a proposal or not immediately after carefully reading the title and abstract, so do not underestimate them.

Again, many busy reviewers will read your title and abstract more carefully. Because scientists are quite busy, there is a tendency to use fast thinking rather than a slow and thoughtful analysis of projects [12]. Usually, after reading the title and the abstract, the reviewer will have already made a decision about whether his or her thumb will be up or down for the proposal. If your title and abstract are well designed, the reviewer will continue reading and will give you several other opportunities to sell your work. Check your title and abstract many times if necessary, and never leave spelling mistakes in them. Spelling mistakes and/or inadequate language at the beginning score much higher in the negativity scale of the reviewer than such mistakes in the middle of the text.

## Rule 10: Organize a Logical Structure and Make the Text More Readable

Your proposal should be concise and impart as much information as possible in the least number of words. After ensuring that your research has precise and feasible objectives, is well contextualized and justified, has a consistent schedule, and is convincingly introduced, it is time to review the text. In addition to the items outlined here (Title, Abstract, Introduction, and so on), you can add other items you find appropriate. Check for a model provided by the funding institution. If no such model is available, take a careful look at successful projects. Try not to be too creative in the way you organize your proposal. You do not need to be a copy machine, but try to respect practices already consolidated. Organize the text in order to make it enjoyable, educational, and accurate [11].

Review your text to find small mistakes that are easy to spot. An excess of easily detectable mistakes suggests laziness. Be careful with the bibliography, which is tedious to organize, because it is very easy to leave mistakes there. The accuracy of references is extremely important because a reader (reviewer) may at any time become curious and check one of them. Give preference to software that automatically organizes references, but also remember that you require a good word processing software program.

Language and semiotics have to be carefully adjusted by many people in the world, and Brazilians are not an exception. Brazilians do not like informal language, so the use of the words “I” or “we” in the text should be avoided. Although Brazilians are not usually direct when speaking, Portuguese has to be transformed into the English style for science texts, using short phrases and sparse punctuation. Discrete humility is important in the text. It is important to find the right balance regarding how you value your work.
